# Supplementary material for: MetaRibo-Seq measures translation in microbiomes
Source: Nat Commun. 2020 Jun 29;11:3268. doi: 10.1038/s41467-020-17081-z (PMC7324362; doi:10.1038/s41467-020-17081-z)
Supplement: Supplementary file 10 — Supplementary Data 7 [file 41467_2020_17081_MOESM10_ESM.zip › File2/Confidence_High_Taxonomy/34981_out.krona.html]

Javascript must be enabled to view this page.

members
magnitude
magnitudeUnassigned
count
unassigned
taxon
rank

34981\_out

10

superkingdom
10
2

10
1239
phylum

class
186801
6

186802
6
order

family
186806
6

6
1730
genus

species

SRS015663\_contig\_number\_4577SRS077294\_contig\_number\_234
2292349
2

species
2292354
4

SRS017247\_contig\_number\_77SRS024075\_contig\_number\_67SRS024492\_contig\_number\_88SRS1041118\_contig\_number\_43

909932
2
class

order
1
1843488

1
909930
family

genus
904
1

SRS063370\_contig\_number\_109

1
1843489
order

family
1
31977

1
39948
genus

1
1955814

SRS024075\_contig\_number\_1940
species

526524
2
class

2
526525
order

2
128827
family

genus
1573535
2

1735
2

SRS1041032\_contig\_number\_8002SRS1041037\_contig\_number\_1
species
